# Population density of the spur-thighed tortoise *Testudo graeca* declines after fire in north-western Africa

**DOI:** 10.1371/journal.pone.0220969

**Published:** 2019-08-16

**Authors:** Brahim Chergui, Roberto C. Rodríguez-Caro, Eva Graciá, Soumia Fahd, Xavier Santos

**Affiliations:** 1 Equipe de Recherche Ecologie, Systématique, Conservation de la Biodiversité, Faculté des Sciences de Tétouan, Université Abdelmalek Essaâdi. BP 2121 El M’Hannech, Tétouan, Morocco; 2 Departamento de Biología Aplicada, Universidad Miguel Hernandez, Elche, Alicante, Spain; 3 Departamento de Ecología, Universidad de Alicante, Alicante, Spain; 4 CIBIO/InBIO (Centro de Investigação em Biodiversidade e Recursos Genéticos da Universidade do Porto). R. Padre Armando Quintas,Vairão, Portugal; INIBIOMA-CONICET, ARGENTINA

## Abstract

Fire is a key ecological process in several biomes worldwide. Over recent decades, human activities (e.g. rural abandonment, monoculture plantations) and global warming are magnifying the risk of fire, with changes in fire intensity and frequency. Here, we offer the first study that examines the impact of fire on the spur-thighed tortoise *Testudo graeca* living in a native cork oak forest and pine plantation in north-western Africa. A total of 44 transects (22 burnt and 22 unburnt) were sampled at 8 sites affected by fires of natural cork oak forest and pine plantation with 8 surveys per site in 2015–2017 (264 hours of sampling effort). Tortoise densities were estimated with line-transect distance sampling. The detection probability of tortoises was higher in burnt (0.915) than unburnt (0.474) transects. The density of tortoises was negatively associated with elevation and declined with fire by c. 50% in both forest types. The negative response of *T*. *graeca* to fire should be considered in conservation planning of this species in north-western Africa in a future scenario of changes in fire regime.

## Introduction

Fire is a widespread process that plays a key role in ecosystem functioning [1]. Both landscape alteration and fire-regime shifts can impact vertebrate populations in the Mediterranean area [2, 3], altering species distribution (extinction or colonization of burnt territories; [4]), and population abundance (population decline or surge; [2]). Animals, being influenced by local environmental conditions, respond differently to fire [5]. That is, some species are early colonizers of recently burnt habitats, while other species are late colonizers and require long-unburnt forests [6–8]. Among reptiles, some species have particular life-history traits that make them vulnerable to fire because they show limited dispersal abilities, delayed reproduction, and low reproductive output, in addition to inhabiting mainly the forest understory [9, 10]. Several studies have indicated that the impact of fire on tortoises is complex [5], since wildfire can directly cause tortoise mortality [11, 12], and can indirectly harm tortoises by fragmenting their habitat and by changing the plant communities that provide these animals both food and shelter [13–15]. The impact of fire on the habitat structure can influence the ability of tortoises to thermoregulate [16]. Additionally, differences in plant cover may change the thermal environment available to tortoises below ground in burrows, where tortoises spend most of their life [17, 18].

Some of their life-history traits can make tortoises especially vulnerable to fire. For example, being slow moving, they have limited ability to escape from flames [19]. Tortoises are also vulnerable to other factors of mortality because of their slow growth, delayed maturity, and high natural mortality both in the egg and at juvenile life stages [20, 21]. Disturbed burnt habitats may also diminish tortoise reproductive output and body condition [12]. These factors can also reduce population recruitment after fire disturbances [5, 22]. Contrary to these general trends, some post-fire habitat shifts can favour tortoise populations. For instance, fire can increase light penetration and temperature by opening the understory, offering opportunities for ectothermic vertebrates to maintain optimal body temperature [23–25]. Fire can also promote vegetative regrowth and foraging opportunities for herbivores such as tortoises [26].

The spur-thighed tortoise (*Testudo graeca*) is a medium-sized species distributed widely in the Mediterranean region [27–31]. This region is characterized by recurrent summer canopy-fire regimes [32]. Negative effects of fire on *T*. *graeca* populations have been reported in Bulgaria [33] and Spain [22, 21, 34]. Land-use changes such as rural depopulation and land abandonment cause fuel accumulation in the forest, and climatic warming are increasing fire intensity and extent in some Mediterranean areas [35]. This fire-regime shift may have several negative consequences for tortoise populations. Moreover, *T*. *graeca* moves slowly, increasing its vulnerability to local extinction, which may be exacerbated by the impact of local changes in habitat quality [36–37].

Here, we analyze the response of *T*. *graeca* to fire in two forest habitats, cork oak forests and pine plantations. Specifically, we seek to identify the factors affecting tortoise density, i.e. forest type (cork oak and pine), fire condition (unburnt and burnt plots), elevation, and structural habitat variables (tree canopy, shrub cover, and bare ground). This is the first field study focused analysing impact of fire in African populations of the spur-thighed tortoise. For this reason, we also discuss conservation measures that are advisable to manage, protect, and recover this endangered species in areas subject to shifts in the fire regime in the coming decades [32].

## Material and methods

### Study species and study area

**Fig 1:**
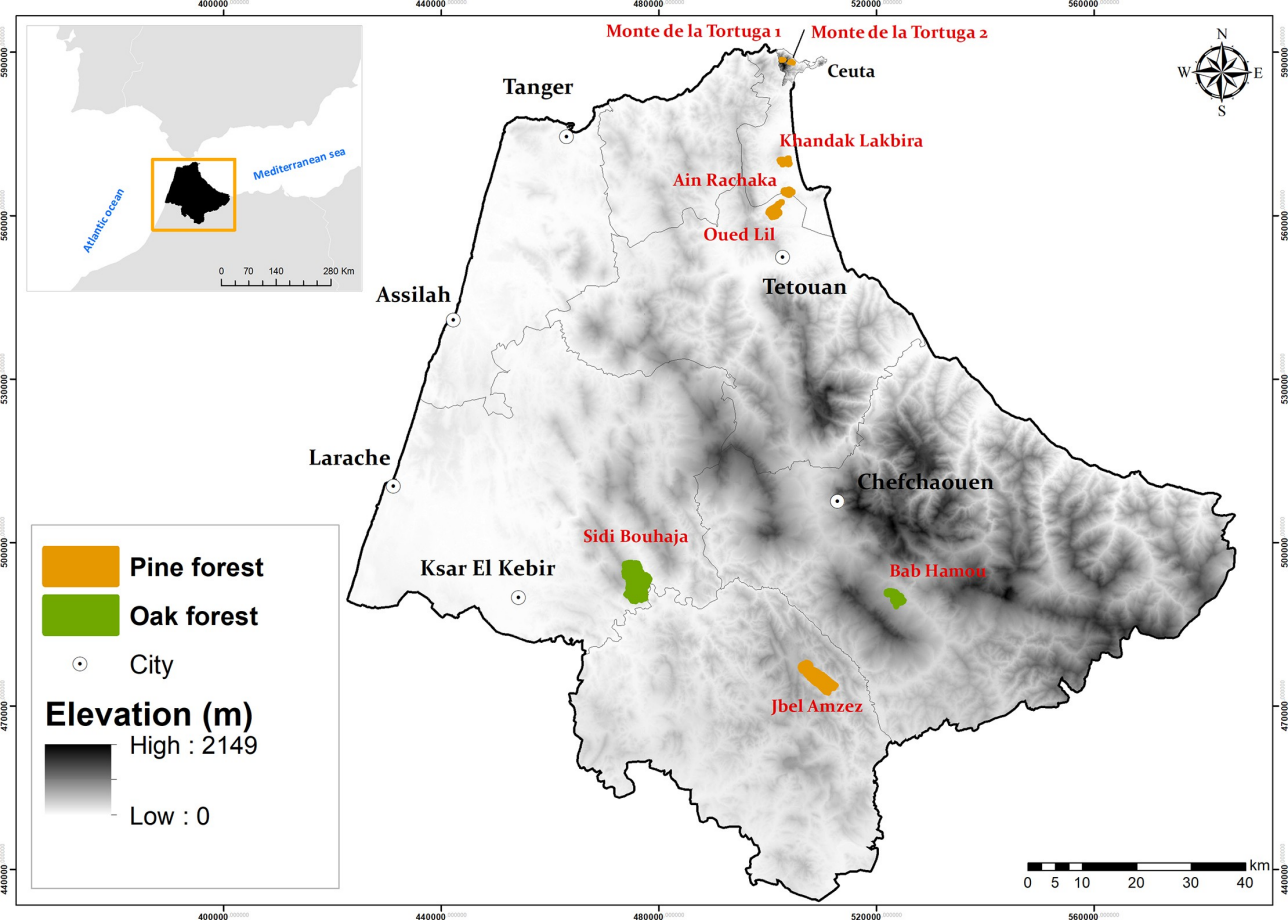
Location of the study sites in the north-western Africa and forest types within the field sites.

The spur-thighed tortoise, *T*. *graeca* is a medium-sized tortoise with an adult body mass ranging in adult individuals from 215 to 880g [38]. Females lay one to four clutches with an average clutch size of 3.5 eggs [39]. Age at maturity varies from 5.8 to 7.6 years in males and 7.7–10.5 years in females [40]. The range of this turtle extends c. 6500 km (east-west) and 1600 km (north-south) [41, 31], with a patchy distribution covering the Mediterranean coastal belt of three continents (Africa, Asia, and Europe), from southern Spain and North Africa to Iran, Asia Minor, and Eastern Europe. The study area is located in north-western Africa, between 35°00′ - 35°55′N and 5°00′ - 6°15′W, covering roughly 12650 km^2^ (Fig 1). The area surveyed lies between 30 and 750 m a.s.l. The climate is typical Mediterranean, with a mean annual temperature of 15°-19° C [42], and annual rainfall ranging between 600 mm and 2000 mm [43]. Patchy vegetation covers 35% of the total area, including natural woodlands and scrublands (84.5%) as well as monocultures (15.5%) [44]. The region is considered very rich and diverse in vegetation cover [45, 46], with natural hardwood forests dominated by cork oak (*Quercus suber*) and holm oak (*Quercus rotundifolia*). Monocultures include 49,124 ha of coniferous trees, mainly maritime pine *Pinus pinaster* and Aleppo pine *Pinus halepensis* [47].

The institution in Morocco that gives scientific permit is the « Haut-Commissariat aux Eaux et Forêts et à la Lutte Contre la Désertification » [HCEFLCD].

### Tortoise surveys

To determine the tortoise population density, we used the line-distance sampling technique [48], laying out 44 transect lines (22 burnt and 22 unburnt transects) located in 8 sites affected by recent fires (2006–2015; see Table 1). The surface area burnt is dissimilar among sites (30–1480 ha; Table 1); however, the location of the burnt transects and the time since fire at each site allowed tortoise recolonization. At each site, burnt and unburnt areas were part of a homogeneous forest without human-made barriers to prevent tortoise movement (the average distance between transects at each site was 1200 m). The forest was composed of cork oak trees (n = 2 sites) and afforested pines (n = 6 sites). At each site, we randomly laid out four to six transects (2–3 burnt and 2–3 unburnt), depending on the size of the burnt area. Transect averaged 733 ± 88 m long and were located at 259 m mean elevation (range 30–718 m), and set at more than 100 m from the fire limits to avoid ecotone effects on tortoise counts.

**Table 1:** Summary of the fire history at the eight sites sampled in north-western Africa. For each study area, the information of the forest type, area burnt (ha), date of fire, elevation (m), time since fire (TSF; in years) when the sampling occurred, and number of tortoises detected in the burnt and unburnt transects.

Site	Date of fire	Burnt area	Forest types	TSF	Elevation	No. of Transects	# *T*. *graeca*	
							burnt	unburnt
Ain Rachaka	04/09/2015	100	Pine plantation	1–2	109	6	10	6
Bab Hamou	22/08/2012	199	Cork oak	3–5	658	6	0	2
Jbel Amezez	2/9/2006	1443	Pine plantation	9–11	531	6	0	2
Khandak lakbira	05/08/2012	110	Pine plantation	3–5	99	6	13	5
Monte de la Tortuga 1	07/09/2014	36	Pine plantation	1–3	154	4	3	0
Monte de la Tortuga 2	06/10/2015	30	Pine plantation	1–2	175	4	1	6
Oued Lile	05/09/2014	286	Pine plantation	1–3	97	6	19	16
Sidi Bouhaja	10/08/2012	1480	Cork oak	3–5	360	6	4	4

Each transect was systematically sampled for 45 min, and visited 8 times for a total of 264 sampling hours (three times in 2015, three times in 2016, and two times in 2017). Transects were sampled by one researcher during spring and autumn, the seasons when tortoises were the most active [49]. Almost all the surveys were conducted by a single researcher (BC), walking during sunny and warm days when temperatures exceeded 20°C and tortoise activity was at its peak [50]. For each tortoise found, the distance along the transect and its perpendicular distance from the transect line were recorded, after which the animal was photographed and released immediately at the point of capture.

We measured three variables that characterized the habitat structure and that served as surrogates of the three vegetation layers: tree canopy (overstory), shrub cover (midstory) and bare ground cover (absence of understory). Along a 700-m line located within the tortoise transect, the tree canopy was measured 50 times with a densitometer mounted on a tripod and oriented towards the four cardinal points, and four canopy measurements were taken. Tree canopy scores were the average of the four cardinal-point measurements [51]. Shrub and bare ground covers were visually estimated on 50 10x10 m quadrats located 15 m apart (25 quadrats on each side of the transect) along the 700-m line. For each tortoise transect, the three structural variables were calculated as the mean value of individual scores on each quadrat.

### Distance sampling analysis

We used distance-sampling models [52], and adopted a two-step modeling procedure [53, 34] to estimate tortoise densities in burnt and unburnt transects from cork oak forest and pine plantation. In the first step, we fitted a detection function to the distance. Truncation of long-distance records is a common practice in distance-sampling methods to improve model fit [52]. Tortoise observations were truncated at > 2.5 m (4% of the tortoises recorded were excluded), as detection probabilities generally fell to 0.1 or lower [48, 34]. Due to the observers’ tendencies to round distance scores, we grouped the detections into five distance intervals (cutpoints: 0, 0.5, 1, 1.5, 2, 2.5m [34]).

We considered six covariates individually: fire condition (burnt and unburnt transects), forest type (cork oak and pine plantation), time since fire (TSF), and three habitat-structure measurements (tree canopy, shrub cover, and bare ground) to evaluate one at a time the fit of the half-normal (HN) key function, and to explore covariate distance-sampling techniques (MCDS) [54, 55] in order to model heterogeneity in detection probabilities. Sampling in different years (2015–2017) can represent different tortoise detectability, given that the vegetation can change according to the time after the fire. Therefore, we considered TSF as covariate in the distance-sampling models. The model selected for inference was that with the lowest Akaike information criterion (AIC, [56]) and its adequacy was assessed using the chi-squared goodness-of-fit test. The best-fitting model was used to estimate density in the second step [34].

In the second step, we related adjusted counts given the detection probability to the covariates that may influence tortoise densities. We used generalized linear mixed models (GLMMs) and a quasi-Poisson distribution to model tortoise density. As fixed effects we used fire condition (burnt or unburnt), forest type (cork oak, pine plantation), the interaction fire x forest type, elevation (m) and the three structural variables (tree canopy, bare ground cover, and shrubs cover). Site and sampling Year nested in site were used as random effects. The GLMMs models were fitted using the glmmPQL function in the MASS package [57]. Since glmmPQL output does not include a deviance component, it is not possible to assess the model’s performance based on AIC criteria. Thus, model selection was done by the backward elimination of terms with no significant effect on estimating tortoise density. Detection-function models were examined with the packages distance and mrds [58, 59]. Detectability models for *T*. *graeca* were built with R software [60]. Multicollinearity was examined by regarding the variance inflation factor (VIF) values of all the factors (car package [61]). Indeed, Time-since-fire proved to be strongly correlated with fire condition (VIF > 5 for both variables). Once TSF was removed from the GLMM models, fire condition had a VIF < 2. The two variables were very correlated because all the unburnt transects had been long unburnt (TSF = 50 years according to the Moroccan data set) whereas the burnt transects ranged from 1 to 5 years since fire except one site that had burnt in 2006. These pronounced TSF differences between burnt and unburnt transects resulted in a high correlation of this variable with the variable fire condition.

## Results

**Fig 2:**
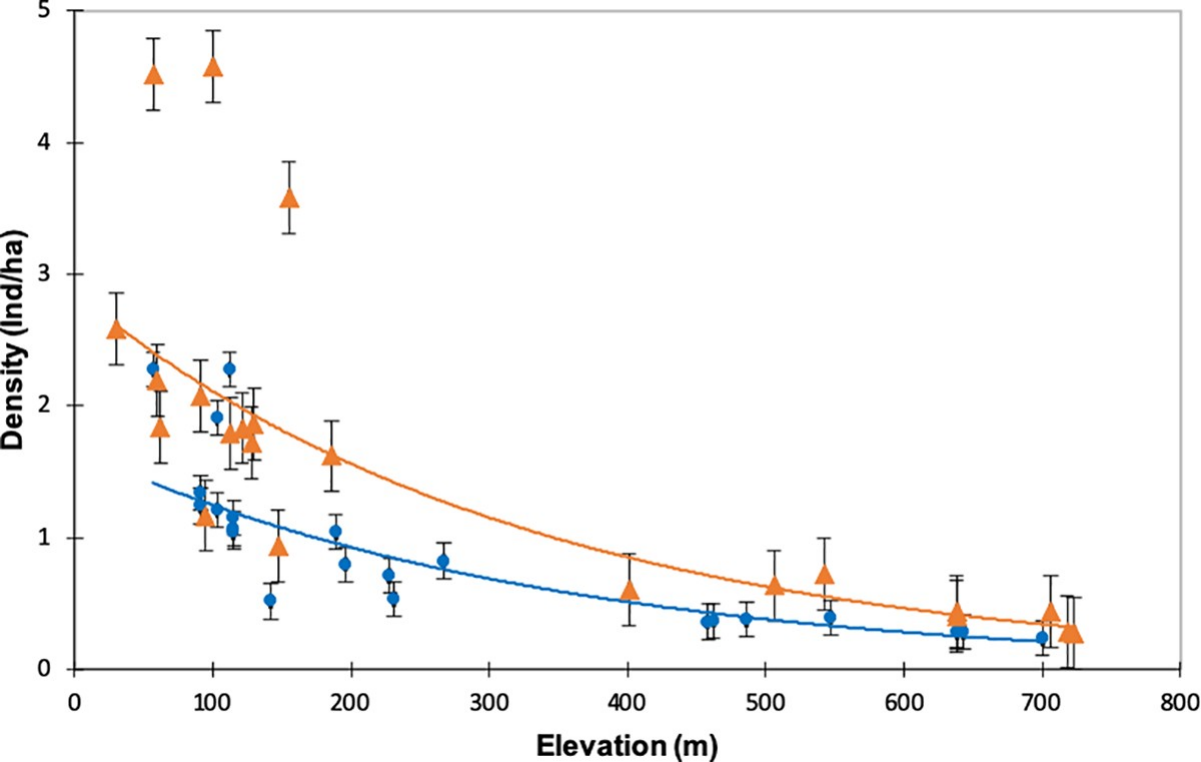
Variation in the density of tortoises (individuals/ha) by fire condition (burnt or unburnt) and elevation (m). Densities derived from the best-fitting quasi-Poisson count model (i.e., Fire + Elevation). Circles estimated mean density of tortoises in burnt transect, triangles mean densities in unburnt transect, and whiskers indicate the 95% confidence intervals.

A total of 91 tortoises were spotted in 44 transects (n = 84 after truncation), the abundance varying from 0 to 16 individuals per transect (Table 1). Additionally, we found four individuals of *T*. *graeca* dead one year after the fire (S1 Fig**)**. Based on the fitted detection function model (step 1), the best model included fire condition as a covariate (Table 2). The detection probability of *T*. *graeca* increased from unburnt (probability = 0.474; 95% CI: 0.393–0.572) to burnt transects (probability = 0.915; 95% CI: 0.692–1.000). Based on p values of GLMM analyses, the best model indicated that tortoise densities were affected by fire condition and elevation (S1 Table). Based on the estimated density of tortoises at each transect (S2 Table), GLMMs indicated that tortoise density significantly declined with fire by around a 50% in both forest types and at higher elevations (Table 3 and Fig 2). At low elevation, the estimated density of tortoises increased with higher values in unburnt transects compared to burnt transects (Fig 2).

**Table 2:** Selection of the best detection function model of tortoises. Six variables that characterize tortoise transects were compared: fire (burnt and unburnt), forest type (cork oak forest and pine plantation forest), time since fire (TSF), tree canopy, shrub cover, and bare ground cover. Each variable was tested using the half-normal (HN) function. Models were ordered by the AIC values. ΔAIC indicates the difference between the chosen model and the specified mode. P value of the Goodness-of-fit (GoF) test.

Key function	Covariates	AIC	ΔAIC	GOF-p
Half normal	**Fire**	**237.444**	0.00	> 0.05
Half normal	TSF	239.502	2.058	> 0.05
Half normal	Bare ground cover	248.495	11.051	0.023
Half normal	–	252.053	14.609	0.031
Half normal	Tree canopy	252.342	14.898	0.014
Half normal	Forest type	253.139	15.695	0.013
Half normal	Shrub cover	253.267	15.823	0.012

**Table 3:** Factors affecting tortoise density. Results of the best generalized linear mixed models (GLMMs) with quasi-Poisson error structure to explain differences in tortoise density. The complete set of candidate models are listed in supplementary materials S1 Table.

Model	Estimate	SE	*t*	*p value*
Intercept	-0.626788	0.3152867	-1.9879	0.0477
Fire	0.515394	0.236677	2.1776	**0.0302**
Elevation	-0.002482	0.000898	-2.7621	**0.0061**

## Discussion

This study evidenced for the first time in Africa the negative responses of *T*. *graeca* populations to fire. Contrary to the estimated density pattern (higher in unburnt transects), the detection probability of *Testudo graeca* was higher in burnt than in unburnt areas. Fire structurally alters the habitat, decreasing the canopy and shrub cover while increasing bare ground [62]. This transformation can enhance detection probability of tortoises driven by the post-fire habitat openness [63].

The most evident result of our study was that tortoise abundance declined from unburnt to burnt transects regardless of the type of forest studied. This negative response appears to be caused by direct mortality during the fire, and also by the unsuitable post-fire habitat conditions. Although we lack estimates for direct mortality in *T*. *graeca*, this has been evidenced by the presence of dead tortoises during the fieldwork. We might expect similar direct mortality rates as those found in tortoise populations of *Testudo* located on the European rim of the western Mediterranean [64, 22, 21]. In the case of *T*. *hermanni*, fire is considered one of the most serious threats, and a direct cause in the population reduction and fragmentation of its distribution [49, 63].

In our study area, the fire regime is characterized by small-sized (usually < 1000 ha burnt) summer fires [32]. These fires tend to cause less mortality in dry regions because of the sparser vegetation cover [65]. Moreover, the recovery of tortoise populations can be rapid in relatively small burnt patches due to recolonization from surrounding unburnt areas (see [66] for other reptiles). Therefore, the unsuitability of the burnt areas related to variation in solar radiation and food availability of (grasses) [67] may be the prime cause of the tortoise population decline after fire. For example, tortoises, being slow-moving animals, are susceptible to dehydration and overheating while moving through open habitats [68]. Moreover, burnt habitats alter the vegetation composition, canopy structure, and shelter abundance, ultimately affecting tortoises negatively [63]. Also, the massive destruction of vegetation probably reduces the availability of shelter, perturbs tortoise thermoregulation, and may generate deleterious chronic stress, causing excessive energy expenditure by tortoises, thereby harming their body condition [11, 69]. The high percentage of bare soil and the substantial post-fire reduction in vegetation cover may intensify predation pressure for tortoises by the absence of adequate shelters [70, 34]. In summary, burnt forests may constitute inadequate habitats for tortoises in north-western Africa due to low food availability and harmful thermal conditions compared to unburnt forests [11, 12, 71]. Unshaded (burnt) habitats do not allow tortoises to maintain body temperature within tolerable physiological limits [72, 73].

The spur-thighed tortoise selects areas with intermediate grass cover and rejects areas with very high or very low cover [74]; thus, the extent of grass cover is a surrogate of food availability for this herbivorous reptile [74]. Thus, tortoises prefer structurally complex habitats during both the activity and aestivation/hibernation seasons [75] and thus move from burnt to unburnt patches immediately following fire (at least in the case of *T*. *hermanni*) [76].

*Testudo graeca* evidenced a high ecological tolerance since its habitat ranges from arid zones in the western of Morocco with mean annual rainfall of about 240 mm [38] to rainy areas in the strait of Gibraltar region with annual rainfall of 800 mm [77]. The abundance of tortoises can be driven by climate (maximum probability of presence occurs from 60 to 180 mm of annual precipitation in eastern Iberia; [78]), and non-climate environmental factors such as lithology, relief, and land uses [79]. However, the estimated density of *T*. *graeca* in north-western Africa declined with elevation in both burnt and unburnt sampled transects, and was not primarily affected by forest type. The distribution of the study sites also influenced the estimated density of tortoises in cork oak and pine plantations, since most pine sites were at lower elevations than were the cork oak stands. The range of terrestrial ectothermic species like *T*. *graeca* is partially limited by environmental factors such as temperature [27], which declines with elevation [80]. Thus, the distribution of *T*. *graeca* is determined by climate and vegetation [27], and also shaped by fire occurrence. The net effect of these environmental differences is hypothesised to cause differences in the total amount of resources available to the tortoise [81].

## Conservation remarks

Our study has shown that forest fires exert a negative impact on *T*. *graeca* populations. This harm may be relevant for *T*. *graeca* conservation at the regional level. Given the slow tortoise recovery after habitat destruction [65], the impact of fire on tortoise populations may continue over the medium and long terms [76]. Moreover, fire frequency and extent is expected to increase along the African rim of the western Mediterranean [32]. The post-fire survival decline of *T*. *gracea* is likely to lead to population crashes in short intervals between fires [22]. Consequently, we expect an overall negative impact of tortoise populations in recurrent fire regimes, as reported for *T*. *hermanni* [10]. In this scenario, management practices such as the maintenance of oak stands within pine plantations should be taken in consideration. This would have an added benefit of reducing fire because oak forests are less flammable than are pine forests [82].

Given the negative impact of fire, the protection of long-unburnt natural oak forest where tortoise density raise, can be an adequate conservation measure at the regional level. Unfortunately, in a future scenario of increasing fire frequency and extension [32], the protection of log-unburnt forests is very difficult due to the remoteness of some forest patches in Morocco. In addition, improving connectivity between forest patches by creating corridors would allow natural tortoise populations to colonize new habitats [83]. In conclusion, conservation planning should consequently focus on measures based on wildfire control, maintenance of a high level of landscape complexity, and prevention of the conversion of scrublands to artificial and agricultural areas to improve the conservation status of the species in the near future [63]. Further investigation is necessary to predict the long-term impacts of fire on this threatened reptile.

## Supporting information

S1 Fig Tortoise dead during the 2015 fire in Ain Rachaka. The picture was taken in November 7^th^, 2015.
(DOCX)

S1 Table Results of the generalized linear mixed models (GLMMs) with quasi-Poisson error structure to select the best model to explain differences in tortoise density. Models were ordered according to their p-values. Multicollinearity was checked with Variance Inflation Factors (VIF) for the variables included in the multivariate model.
(DOCX)

S2 Table Tortoise density estimates (individuals/ha) in the sampled transects at north-western Africa according to the best model (fire + elevation). Density values are adjusted according to the detection probability.
(DOCX)

S1 Dataset Surveys data. This matrix includes details of each tortoise observation such as the date, transect, site and distance from the observer.
(XLS)
