# Supplementary figures and images for: Population density of the spur-thighed tortoise Testudo graeca declines after fire in north-western Africa
Source: PLoS One. 2019 Aug 16;14(8):e0220969. doi: 10.1371/journal.pone.0220969 (PMC6697351; doi:10.1371/journal.pone.0220969)

**S1 Fig. Tortoise dead during the 2015 fire in Ain Rachaka**. The picture was taken in November 7th, 2015.
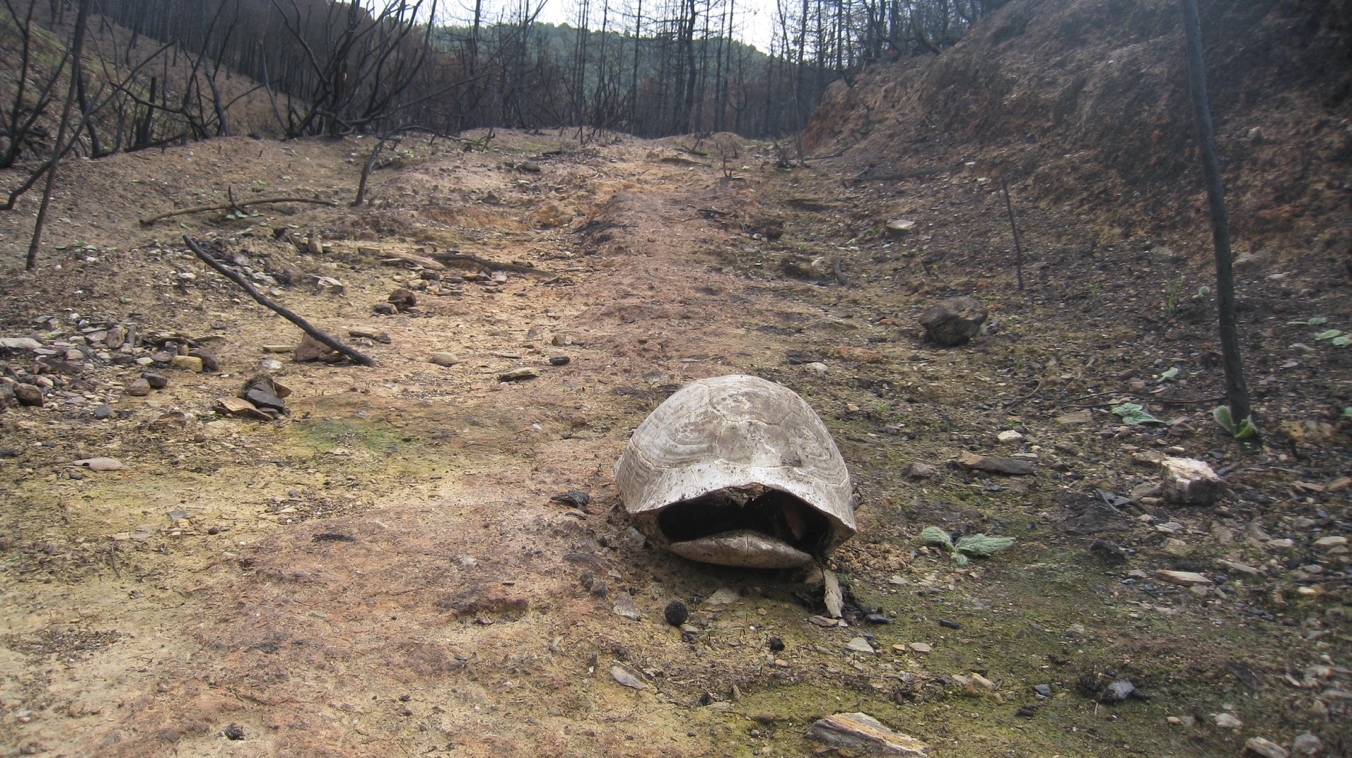

Supplement: S1 Fig — The picture was taken in November 7th, 2015. (DOCX) [file pone.0220969.s001.docx]
