# Supplementary material for: Population density of the spur-thighed tortoise Testudo graeca declines after fire in north-western Africa
Source: PLoS One. 2019 Aug 16;14(8):e0220969. doi: 10.1371/journal.pone.0220969 (PMC6697351; doi:10.1371/journal.pone.0220969)
Supplement: S1 Table — Models were ordered according to their p-values. Multicollinearity was checked with Variance Inflation Factors (VIF) for the variables included in the multivariate model. (DOCX) [file pone.0220969.s002.docx]

**S1 Table. Results of the generalized linear mixed models (GLMMs) with quasi-Poisson error** structure to select the best model to explain differences in tortoise density. Models were ordered according to their p-values. Multicollinearity was checked with Variance Inflation Factors (VIF) for the variables included in the multivariate model.

| **Model** | **Estimate** | **SE** | ***t*** | ***p value*** | **VIF** |
| --- | --- | --- | --- | --- | --- |
| **Model 1: Fire + elevation** | | | | | |
| Fire | 0.515394 | 0.236677 | 2.1776 | **0.0302** | 1.003 |
| Elevation | -0.002482 | 0.000898 | -2.7621 | **0.0061** | 1.003 |
| **Model 2: Fire + forest type + elevation** | | | | | |
| Fire | 0.518762 | 0.236545 | 2.1930 | **0.0290** | 1.002 |
| Forest type | -0.366058 | 0.553811 | -0.6609 | ns | 1.250 |
| Elevation | -0.002196 | 0.001008 | -2.1767 | **0.0302** | 1.253 |
| **Model 3: Fire + forest type + elevation + tree canopy**  **+ bare ground + shrubs cover** | | | | | |
| Fire | 0.521341 | 0.248083 | 2.1014 | **0.0364** | 1.098 |
| Forest type | -0.390608 | 0.544095 | -0.7179 | ns | 1.305 |
| Elevation | -0.002198 | 0.000987 | -2.2270 | **0.0266** | 1.270 |
| Tree canopy | 0.002093 | 0.006056 | 0.3456 | ns | 1.215 |
| Bare ground | -0.003372 | 0.010727 | -0.3143 | ns | 1.278 |
| Shrubs cover | 0.011438 | 0.014540 | 0.7866 | ns | 1.138 |
| **Model 4: Fire * forest type + elevation** | | | | | |
| Fire | 0.4544562 | 0.2518449 | 1.8045 | 0.0721 | 1.116 |
| Forest type | -0.7634114 | 0.8035134 | -0.9500 | ns | 2.612 |
| Elevation | -0.0021913 | 0.0010006 | -2.1899 | **0.0292** | 1.242 |
| Fire * Forest type | 0.5860735 | 0.7983154 | 0.7341 | ns | 2.506 |
| **Model 5: Forest type + fire** | | | | | |
| Fire | 0.5244644 | 0.2290751 | 2.2894 | **0.0227** | 1.000 |
| Forest type | -0.9284820 | 0.6347859 | -1.4626 | ns | 1.000 |
| **Model 6: Forest type + elevation** | | | | | |
| Forest type | -0.3266219 | 0.5594144 | -0.5838 | ns | 1.264 |
| Elevation | -0.0024358 | 0.0010751 | -2.2657 | **0.0241** | 1.264 |
