# Supplementary material for: Population density of the spur-thighed tortoise Testudo graeca declines after fire in north-western Africa
Source: PLoS One. 2019 Aug 16;14(8):e0220969. doi: 10.1371/journal.pone.0220969 (PMC6697351; doi:10.1371/journal.pone.0220969)
Supplement: S2 Table — Density values are adjusted according to the detection probability. (DOCX) [file pone.0220969.s003.docx]

**S2 Table. Tortoise density estimates (individuals/ha) in the sampled transects at north-western Africa** according to the best model (fire + elevation)**.** Density values are adjusted according to the detection probability.

| **Study site** | **Transect**  **Code** | **Fire condition** | **Altitude (m)** | **Density**  **Ind./ha** |
| --- | --- | --- | --- | --- |
| **Ain Rachaka** | AIR1B | Burnt | 115 | 1.044 |
|  | AIR1U | Unburnt | 128 | 1.719 |
|  | AIR2B | Burnt | 115 | 1.069 |
|  | AIR2U | Unburnt | 60 | 2.190 |
|  | AIR3B | Burnt | 115 | 1.148 |
|  | AIR3U | Unburnt | 121 | 1.831 |
| **Bab Hamou** | BH1B | Burnt | 700 | 0.232 |
|  | BH1U | Unburnt | 706 | 0.437 |
|  | BH2B | Burnt | 638 | 0.285 |
|  | BH2U | Unburnt | 542 | 0.720 |
|  | BH3B | Burnt | 643 | 0.279 |
|  | BH3U | Unburnt | 718 | 0.281 |
| **Jbel Amzez** | JAZ1B | Burnt | 462 | 0.370 |
|  | JAZ1U | Unburnt | 506 | 0.635 |
|  | JAZ2B | Burnt | 458 | 0.361 |
|  | JAZ2U | Unburnt | 638 | 0.437 |
|  | JAZ3B | Burnt | 486 | 0.374 |
|  | JAZ3U | Unburnt | 638 | 0.406 |
| **Khandak Lakbira** | KL1B | Burnt | 91 | 1.239 |
|  | KL1U | Unburnt | 186 | 1.620 |
|  | KL2B | Burnt | 91 | 1.342 |
|  | KL2U | Unburnt | 91 | 2.073 |
|  | KL3B | Burnt | 103 | 1.208 |
|  | KL3U | Unburnt | 30 | 2.589 |
| **Monte de la Tortuga1** | MN1B1 | Burnt | 231 | 0.535 |
|  | MN1B2 | Burnt | 142 | 0.516 |
|  | MN1U1 | Unburnt | 95 | 1.167 |
|  | MN1U2 | Unburnt | 147 | 0.938 |
| **Monte de la Tortuga 2** | MN2B1 | Burnt | 189 | 1.039 |
|  | MN2B2 | Burnt | 267 | 0.823 |
|  | MN2U1 | Unburnt | 113 | 1.791 |
|  | MN2U2 | Unburnt | 130 | 1.861 |
| **Oued Lil** | OL1B | Burnt | 57 | 2.276 |
|  | OL1U | Unburnt | 57 | 4.515 |
|  | OL2B | Burnt | 112 | 2.275 |
|  | OL2U | Unburnt | 155 | 3.580 |
|  | OL3B | Burnt | 103 | 1.906 |
|  | OL3U | Unburnt | 100 | 4.577 |
| **Sidi Bouhaja** | SB1B | Burnt | 228 | 0.712 |
|  | SB1U | Unburnt | 62 | 1.838 |
|  | SB2B | Burnt | 196 | 0.797 |
|  | SB2U | Unburnt | 401 | 0.605 |
|  | SB3B | Burnt | 547 | 0.393 |
|  | SB3U | Unburnt | 723 | 0.271 |
